# (2*Z*,4*E*)-1-(5-Fluoro-2-hy­droxy­phen­yl)-5-(4-fluoro­phen­yl)-3-hy­droxy­penta-2,4-dien-1-one

**DOI:** 10.1107/S1600536813033564

**Published:** 2013-12-24

**Authors:** Jing-Wei Chen, Zhuo He, Zhen Wu, Mei-Juan Fang, Hua Fang

**Affiliations:** a The Key Laboratory for Chemical Biology of Fujian Province, Xiamen University, Xiamen 361005, People’s Republic of China; b School of Pharmaceutical Sciences, Xiamen University, Sounth Xiang-An Road, Xiamen 361100, People’s Republic of China; c State Ocean Adm, Inst Oceanog 3, Xiamen 361005, People’s Republic of China

## Abstract

In the title mol­ecule, C_17_H_12_F_2_O_3_, the dihedral angle between the benzene rings is 8.6 (2)°. In the crystal, two pairs of O—H⋯O hydrogen bonds connect the mol­ecules into inversion dimers. In addition, weak C—H⋯F hydrogen bonds link the dimers into a two-dimensional network parallel to (10-4). The carbonyl O atom is an acceptor for two weak intra­molecular hydrogen bonds.

## Related literature   

For the biological activities of chalcones, see: Meng *et al.* (2007 ▶); Schobert *et al.* (2009 ▶). For the synthesis, see: Baker (1933 ▶); Mahal & Venkataraman (1934 ▶). For a related structure, see: Fun *et al.* (2012 ▶).
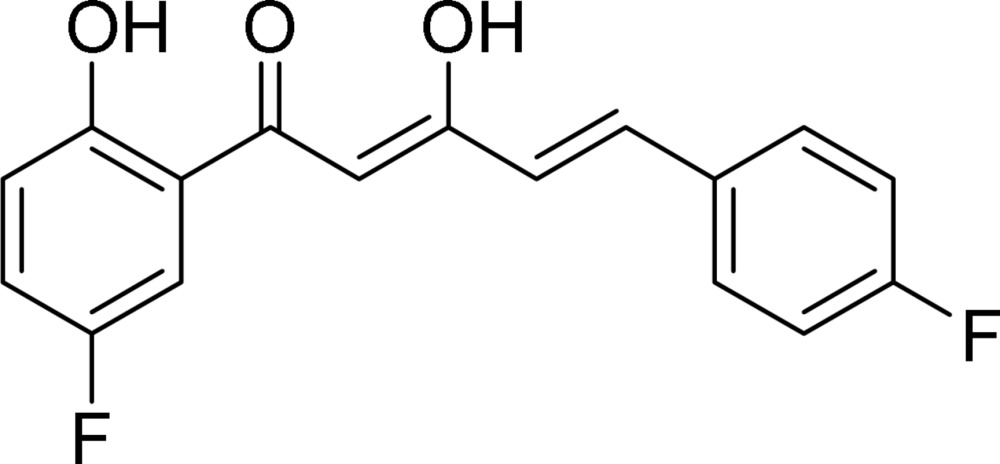


## Experimental   

### 

#### Crystal data  

C_17_H_12_F_2_O_3_
*M* *_r_* = 302.27
Monoclinic, 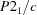

*a* = 6.8275 (18) Å
*b* = 14.004 (4) Å
*c* = 14.267 (4) Å
β = 91.293 (5)°
*V* = 1363.8 (7) Å^3^
*Z* = 4
Mo *K*α radiation
μ = 0.12 mm^−1^
*T* = 173 K
0.32 × 0.23 × 0.18 mm

#### Data collection  

Bruker APEX CCD diffractometer
Absorption correction: multi-scan (*SADABS*; Bruker, 2001 ▶) *T*_min_ = 0.963, *T*_max_ = 0.979
6787 measured reflections
2392 independent reflections
2040 reflections with *I* > 2σ(*I*)
*R*_int_ = 0.028

#### Refinement  

*R*[*F* ^2^ > 2σ(*F* ^2^)] = 0.053
*wR*(*F* ^2^) = 0.147
*S* = 1.08
2392 reflections
199 parameters
H-atom parameters constrained
Δρ_max_ = 0.21 e Å^−3^
Δρ_min_ = −0.29 e Å^−3^

### 

Data collection: *SMART* (Bruker, 2001 ▶); cell refinement: *SAINT* (Bruker, 2001 ▶); data reduction: *SAINT* ; program(s) used to solve structure: *SHELXS97* (Sheldrick, 2008 ▶); program(s) used to refine structure: *SHELXL97* (Sheldrick, 2008 ▶); molecular graphics: *SHELXTL* (Sheldrick, 2008 ▶) and *PLATON* (Spek, 2009 ▶); software used to prepare material for publication: *SHELXL97*.

## Supplementary Material

Crystal structure: contains datablock(s) I. DOI: 10.1107/S1600536813033564/lh5672sup1.cif

Structure factors: contains datablock(s) I. DOI: 10.1107/S1600536813033564/lh5672Isup2.hkl

Supporting information file. DOI: 10.1107/S1600536813033564/lh5672Isup3.cml

Additional supporting information:  crystallographic information; 3D view; checkCIF report

## Figures and Tables

**Table 1:** Hydrogen-bond geometry (Å, °)

*D*—H⋯*A*	*D*—H	H⋯*A*	*D*⋯*A*	*D*—H⋯*A*
O1—H1*A*⋯O2	0.82	1.84	2.558 (2)	145
O1—H1*A*⋯O3^i^	0.82	2.55	3.145 (2)	130
O3—H3*A*⋯O2	0.82	1.81	2.532 (2)	146
O3—H3*A*⋯O2^i^	0.82	2.38	2.856 (2)	118
C10—H10*A*⋯F2^ii^	0.93	2.51	3.326 (3)	147

Symmetry codes: (i) 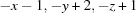; (ii) 
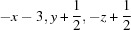
.

**Fig. 1.**
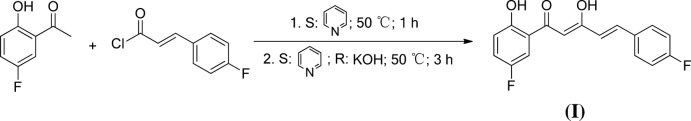
The reaction scheme.

**Fig. 2.**
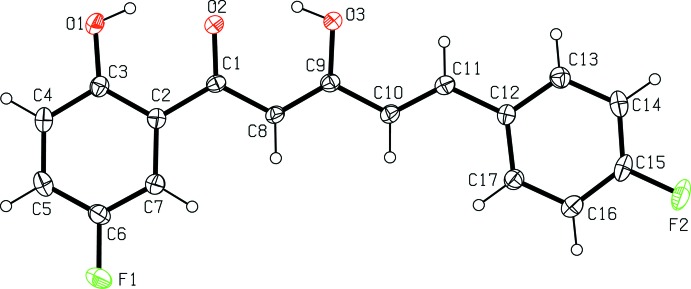
The molecular structure of (I) with displacement ellipsoids drawn at the 30% probability level and H atoms drawn as small spheres of arbitrary radii.

**Fig. 3.**
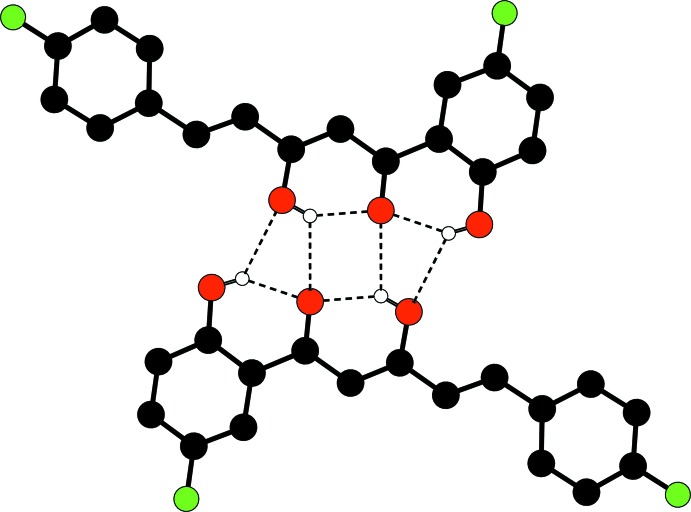
An inversion dimers of (I). Hydrogen bonds are shown as dashed lines.

**Fig. 4.**
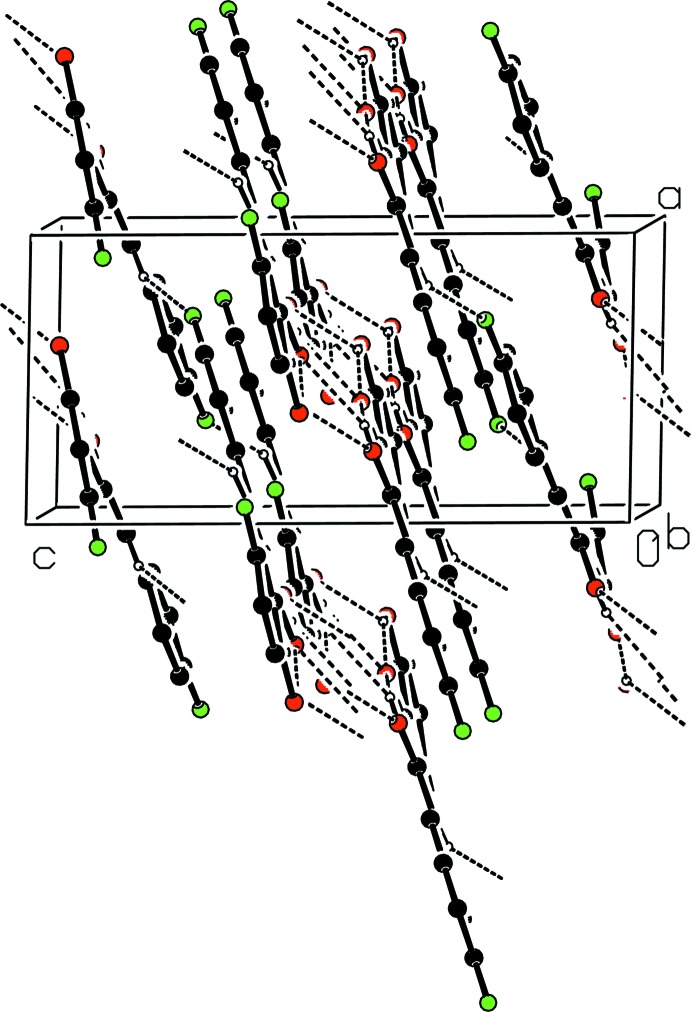
Part of the crystal structure with hydrogen bonds shown as dashed lines.

## supplementary crystallographic information

### 1. Comment

Dibenzoylmethane (1,3-Diphenyl-propane-1,3-dione, DBM) and chalcones have been found to exhibit a variety of biological activities, such as anti-tumor, anti-inflammatory, antibacterial, anti-parasitic, anti-oxidation and anti-viral effect (Schobert *et al.*, 2009; Meng *et al.*, 2007). Recently, we carried out to synthesis a series of (*E*)-1,5-diphenylpent-4-ene-1,3-dione derivatives. These compounds have keto-enol tautomerism, however, the enol form predominates. The dihedral angle between the planes of the phenyl rings (C2–C7) and (C12–C17) is 8.6 (3) °. Bond lengths and angles in (I, Fig. 2) are agreement with values reported for a similar compound (Fun *et al.*, 2012). In the crystal, two pairs of O—H···O hydrogen bonds connect the molecules into inversion dimers (Fig. 3). In addition, weak C—H···F hydrogen bonds link dimers into a two-dimensional network parallel to (104) (Fig. 4). The oxygen atom of the –C═O group is an acceptor for two intramolecular hydrogen bonds (Fig. 2).

### 2. Experimental

The reaction scheme is shown in Fig. 1. A reaction mixture of (*E*)-3-(4-fluorophenyl) acryloyl chloride (0.37 g, 2.0 mmol) and 1-(5-fluoro-2-hydroxyphenyl)ethanone (0.31 g, 2.0 mmol) in pyridine (10 ml) was stirred for 1 h at 323K (Baker *et al.*, 1933). The mixture was then neutralized with dilute hydrochloric acid, and extracted with ethyl acetate (3×15 ml). The organic phase was concentrated under vacuum to obtain a slurry residue to which was added pyridine (10 ml) and potassium hydroxide (0.21 g, 1.5 mmol). The solution was stirred for 3 h at 323K. The mixture was then neutralized with dilute hydrochloric acid, and extracted with ethyl acetate (3×15 ml). The organic phase was dried over anhydrous MgSO_4_ and concentrated under vacuum to obtain a slurry residue. The residue was purified by chromatography using petroleum ether and ethyl acetate (v:v=5:1) as eluent to give a light yellow solid (Mahal & Venkataraman, 1934). Single crystals were obtained by crystallization of a petroleum ether and ethyl acetate (v:v=1:4) solution of the title compound.

### 3. Refinement

All H atoms were placed in geometrically idealized positions and treated as riding on their parent atoms, with C—H = 0.93 (aromatic) or 0.98 (CH), O—H = 0.82 Å and isotropic displacement parameters for were set at *U*_iso_(H) = *x U*_eq_ (carried atom), with *x* = 1.5 for hydroxy, and *x* = 1.2 for methyne.

### Crystal data

**Table:**

C_17_H_12_F_2_O_3_	*F*(000) = 624
*M**_r_* = 302.27	*D*_x_ = 1.472 Mg m^−^^3^
Monoclinic, *P*2_1_/*c*	Mo *K*α radiation, λ = 0.71073 Å
Hall symbol: -P 2ybc	Cell parameters from 2113 reflections
*a* = 6.8275 (18) Å	θ = 1.6–27.3°
*b* = 14.004 (4) Å	µ = 0.12 mm^−^^1^
*c* = 14.267 (4) Å	*T* = 173 K
β = 91.293 (5)°	Block, yellow
*V* = 1363.8 (7) Å^3^	0.32 × 0.23 × 0.18 mm
*Z* = 4	

### Data collection

**Table:**

Bruker APEX CCD diffractometer	2392 independent reflections
Radiation source: fine-focus sealed tube	2040 reflections with *I* > 2σ(*I*)
Graphite monochromator	*R*_int_ = 0.028
φ and ω scan	θ_max_ = 25.0°, θ_min_ = 2.0°
Absorption correction: multi-scan (*SADABS*; Bruker, 2001)	*h* = −8→8
*T*_min_ = 0.963, *T*_max_ = 0.979	*k* = −16→16
6787 measured reflections	*l* = −12→16

### Refinement

**Table:**

Refinement on *F*^2^	Primary atom site location: structure-invariant direct methods
Least-squares matrix: full	Secondary atom site location: difference Fourier map
*R*[*F*^2^ > 2σ(*F*^2^)] = 0.053	Hydrogen site location: inferred from neighbouring sites
*wR*(*F*^2^) = 0.147	H-atom parameters constrained
*S* = 1.08	*w* = 1/[σ^2^(*F*_o_^2^) + (0.073*P*)^2^ + 0.3861*P*] where *P* = (*F*_o_^2^ + 2*F*_c_^2^)/3
2392 reflections	(Δ/σ)_max_ < 0.001
199 parameters	Δρ_max_ = 0.21 e Å^−^^3^
0 restraints	Δρ_min_ = −0.29 e Å^−^^3^

### Special details

**Table:**

Geometry. All e.s.d.'s (except the e.s.d. in the dihedral angle between two l.s. planes) are estimated using the full covariance matrix. The cell e.s.d.'s are taken into account individually in the estimation of e.s.d.'s in distances, angles and torsion angles; correlations between e.s.d.'s in cell parameters are only used when they are defined by crystal symmetry. An approximate (isotropic) treatment of cell e.s.d.'s is used for estimating e.s.d.'s involving l.s. planes.
Refinement. Refinement of *F*^2^ against ALL reflections. The weighted *R*-factor *wR* and goodness of fit *S* are based on *F*^2^, conventional *R*-factors *R* are based on *F*, with *F* set to zero for negative *F*^2^. The threshold expression of *F*^2^ > σ(*F*^2^) is used only for calculating *R*-factors(gt) *etc*. and is not relevant to the choice of reflections for refinement. *R*-factors based on *F*^2^ are statistically about twice as large as those based on *F*, and *R*- factors based on ALL data will be even larger.

### Fractional atomic coordinates and isotropic or equivalent isotropic displacement parameters (Å^2^)

**Table:**

	*x*	*y*	*z*	*U*_iso_*/*U*_eq_	
F1	−1.0830 (2)	1.43310 (10)	0.38367 (11)	0.0579 (5)	
F2	−1.6953 (2)	0.59884 (10)	0.25050 (10)	0.0577 (5)	
O3	−0.7476 (2)	0.93089 (10)	0.42390 (11)	0.0383 (4)	
H3A	−0.6598	0.9675	0.4404	0.057*	
O2	−0.5856 (2)	1.09111 (10)	0.45331 (11)	0.0348 (4)	
O1	−0.3972 (2)	1.24886 (10)	0.46202 (11)	0.0391 (4)	
H1A	−0.4108	1.1908	0.4649	0.059*	
C16	−1.5714 (3)	0.75182 (16)	0.27847 (15)	0.0371 (5)	
H16A	−1.6915	0.7780	0.2602	0.045*	
C6	−0.9127 (3)	1.38522 (15)	0.40188 (16)	0.0371 (5)	
C9	−0.9017 (3)	0.98066 (14)	0.39432 (13)	0.0276 (5)	
C1	−0.7404 (3)	1.13263 (14)	0.42564 (13)	0.0258 (5)	
C7	−0.9178 (3)	1.28835 (15)	0.40533 (15)	0.0328 (5)	
H7A	−1.0352	1.2559	0.3952	0.039*	
C5	−0.7442 (4)	1.43672 (16)	0.41622 (16)	0.0401 (6)	
H5A	−0.7455	1.5030	0.4127	0.048*	
C13	−1.2171 (3)	0.67182 (15)	0.33270 (14)	0.0336 (5)	
H13A	−1.0979	0.6447	0.3511	0.040*	
C8	−0.9043 (3)	1.07757 (14)	0.39450 (14)	0.0287 (5)	
H8A	−1.0170	1.1090	0.3736	0.034*	
C3	−0.5709 (3)	1.28979 (15)	0.44117 (14)	0.0297 (5)	
C14	−1.3700 (4)	0.61349 (16)	0.30486 (16)	0.0400 (6)	
H14A	−1.3558	0.5474	0.3041	0.048*	
C11	−1.0733 (3)	0.83020 (14)	0.36333 (14)	0.0287 (5)	
H11A	−0.9613	0.7987	0.3852	0.034*	
C2	−0.7438 (3)	1.23770 (14)	0.42429 (13)	0.0271 (5)	
C17	−1.4183 (3)	0.80885 (16)	0.30625 (15)	0.0331 (5)	
H17A	−1.4352	0.8747	0.3068	0.040*	
C10	−1.0668 (3)	0.92464 (14)	0.36235 (14)	0.0294 (5)	
H10A	−1.1766	0.9571	0.3394	0.035*	
C15	−1.5432 (3)	0.65543 (17)	0.27832 (15)	0.0385 (6)	
C12	−1.2379 (3)	0.77070 (14)	0.33372 (13)	0.0265 (5)	
C4	−0.5740 (3)	1.38854 (15)	0.43586 (16)	0.0374 (6)	
H4A	−0.4585	1.4226	0.4458	0.045*	

### Atomic displacement parameters (Å^2^)

**Table:**

	*U*^11^	*U*^22^	*U*^33^	*U*^12^	*U*^13^	*U*^23^
F1	0.0450 (9)	0.0381 (8)	0.0906 (12)	0.0127 (6)	0.0004 (8)	0.0083 (8)
F2	0.0554 (10)	0.0552 (9)	0.0619 (10)	−0.0309 (7)	−0.0138 (7)	−0.0001 (7)
O3	0.0275 (8)	0.0281 (8)	0.0588 (11)	0.0005 (6)	−0.0106 (7)	−0.0042 (7)
O2	0.0258 (8)	0.0298 (8)	0.0483 (9)	−0.0013 (6)	−0.0062 (7)	0.0013 (7)
O1	0.0295 (9)	0.0346 (8)	0.0530 (10)	−0.0088 (7)	−0.0058 (7)	0.0058 (7)
C16	0.0306 (12)	0.0444 (13)	0.0363 (13)	−0.0028 (10)	0.0002 (9)	−0.0039 (10)
C6	0.0374 (13)	0.0306 (11)	0.0434 (13)	0.0058 (10)	0.0033 (10)	0.0042 (10)
C9	0.0268 (11)	0.0301 (11)	0.0258 (11)	0.0005 (9)	−0.0012 (8)	0.0017 (8)
C1	0.0235 (10)	0.0308 (11)	0.0231 (10)	0.0005 (8)	0.0001 (8)	0.0016 (8)
C7	0.0307 (12)	0.0318 (11)	0.0358 (12)	−0.0020 (9)	0.0008 (9)	0.0010 (9)
C5	0.0508 (15)	0.0260 (11)	0.0438 (14)	−0.0027 (10)	0.0066 (11)	0.0006 (10)
C13	0.0369 (13)	0.0324 (11)	0.0313 (12)	−0.0010 (9)	0.0002 (9)	0.0019 (9)
C8	0.0251 (11)	0.0297 (11)	0.0310 (11)	−0.0015 (8)	−0.0066 (8)	0.0042 (9)
C3	0.0307 (12)	0.0315 (11)	0.0269 (11)	−0.0046 (9)	0.0012 (9)	0.0016 (9)
C14	0.0535 (15)	0.0282 (11)	0.0383 (13)	−0.0097 (11)	0.0018 (11)	−0.0012 (10)
C11	0.0271 (12)	0.0333 (11)	0.0257 (11)	0.0009 (9)	0.0005 (8)	−0.0027 (9)
C2	0.0305 (11)	0.0284 (11)	0.0224 (10)	−0.0028 (9)	0.0023 (8)	0.0013 (8)
C17	0.0296 (12)	0.0331 (11)	0.0367 (12)	−0.0009 (9)	0.0014 (9)	−0.0043 (9)
C10	0.0274 (11)	0.0316 (11)	0.0290 (11)	−0.0011 (9)	−0.0051 (9)	0.0000 (9)
C15	0.0398 (14)	0.0441 (13)	0.0317 (12)	−0.0205 (11)	−0.0008 (10)	−0.0013 (10)
C12	0.0299 (11)	0.0291 (10)	0.0206 (10)	−0.0032 (9)	0.0018 (8)	−0.0019 (8)
C4	0.0413 (14)	0.0312 (11)	0.0397 (13)	−0.0108 (10)	0.0026 (10)	−0.0030 (10)

### Geometric parameters (Å, º)

**Table:**

F1—C6	1.362 (2)	C5—C4	1.368 (3)
F2—C15	1.358 (2)	C5—H5A	0.9300
O3—C9	1.323 (2)	C13—C14	1.377 (3)
O3—H3A	0.8200	C13—C12	1.392 (3)
O2—C1	1.262 (2)	C13—H13A	0.9300
O1—C3	1.345 (2)	C8—H8A	0.9300
O1—H1A	0.8200	C3—C4	1.385 (3)
C16—C15	1.363 (3)	C3—C2	1.404 (3)
C16—C17	1.367 (3)	C14—C15	1.366 (3)
C16—H16A	0.9300	C14—H14A	0.9300
C6—C7	1.358 (3)	C11—C10	1.323 (3)
C6—C5	1.369 (3)	C11—C12	1.454 (3)
C9—C8	1.357 (3)	C11—H11A	0.9300
C9—C10	1.439 (3)	C17—C12	1.391 (3)
C1—C8	1.422 (3)	C17—H17A	0.9300
C1—C2	1.472 (3)	C10—H10A	0.9300
C7—C2	1.404 (3)	C4—H4A	0.9300
C7—H7A	0.9300		
			
C9—O3—H3A	109.5	O1—C3—C2	123.41 (18)
C3—O1—H1A	109.5	C4—C3—C2	119.84 (19)
C15—C16—C17	118.1 (2)	C15—C14—C13	118.1 (2)
C15—C16—H16A	120.9	C15—C14—H14A	121.0
C17—C16—H16A	120.9	C13—C14—H14A	121.0
C7—C6—F1	118.4 (2)	C10—C11—C12	126.55 (19)
C7—C6—C5	122.9 (2)	C10—C11—H11A	116.7
F1—C6—C5	118.66 (19)	C12—C11—H11A	116.7
O3—C9—C8	122.42 (18)	C3—C2—C7	118.35 (18)
O3—C9—C10	115.18 (17)	C3—C2—C1	120.26 (18)
C8—C9—C10	122.40 (18)	C7—C2—C1	121.38 (18)
O2—C1—C8	119.71 (18)	C16—C17—C12	121.6 (2)
O2—C1—C2	118.55 (17)	C16—C17—H17A	119.2
C8—C1—C2	121.72 (18)	C12—C17—H17A	119.2
C6—C7—C2	119.3 (2)	C11—C10—C9	124.61 (19)
C6—C7—H7A	120.4	C11—C10—H10A	117.7
C2—C7—H7A	120.4	C9—C10—H10A	117.7
C4—C5—C6	118.6 (2)	F2—C15—C16	118.1 (2)
C4—C5—H5A	120.7	F2—C15—C14	118.8 (2)
C6—C5—H5A	120.7	C16—C15—C14	123.1 (2)
C14—C13—C12	121.1 (2)	C17—C12—C13	117.98 (19)
C14—C13—H13A	119.5	C17—C12—C11	122.39 (18)
C12—C13—H13A	119.5	C13—C12—C11	119.63 (19)
C9—C8—C1	122.19 (19)	C5—C4—C3	121.0 (2)
C9—C8—H8A	118.9	C5—C4—H4A	119.5
C1—C8—H8A	118.9	C3—C4—H4A	119.5
O1—C3—C4	116.75 (18)		
			
F1—C6—C7—C2	−179.82 (18)	C8—C1—C2—C7	9.5 (3)
C5—C6—C7—C2	0.0 (4)	C15—C16—C17—C12	0.0 (3)
C7—C6—C5—C4	−0.7 (4)	C12—C11—C10—C9	178.58 (19)
F1—C6—C5—C4	179.0 (2)	O3—C9—C10—C11	1.7 (3)
O3—C9—C8—C1	0.7 (3)	C8—C9—C10—C11	−178.0 (2)
C10—C9—C8—C1	−179.63 (18)	C17—C16—C15—F2	−179.84 (19)
O2—C1—C8—C9	0.8 (3)	C17—C16—C15—C14	0.2 (4)
C2—C1—C8—C9	179.13 (19)	C13—C14—C15—F2	179.86 (19)
C12—C13—C14—C15	−0.1 (3)	C13—C14—C15—C16	−0.2 (4)
O1—C3—C2—C7	178.50 (19)	C16—C17—C12—C13	−0.3 (3)
C4—C3—C2—C7	−2.1 (3)	C16—C17—C12—C11	179.90 (19)
O1—C3—C2—C1	−2.1 (3)	C14—C13—C12—C17	0.3 (3)
C4—C3—C2—C1	177.29 (19)	C14—C13—C12—C11	−179.87 (19)
C6—C7—C2—C3	1.4 (3)	C10—C11—C12—C17	−4.8 (3)
C6—C7—C2—C1	−177.92 (19)	C10—C11—C12—C13	175.4 (2)
O2—C1—C2—C3	8.5 (3)	C6—C5—C4—C3	0.1 (3)
C8—C1—C2—C3	−169.85 (18)	O1—C3—C4—C5	−179.18 (19)
O2—C1—C2—C7	−172.14 (18)	C2—C3—C4—C5	1.3 (3)

### Hydrogen-bond geometry (Å, º)

**Table:**

*D*—H···*A*	*D*—H	H···*A*	*D*···*A*	*D*—H···*A*
O1—H1*A*···O2	0.82	1.84	2.558 (2)	145
O1—H1*A*···O3^i^	0.82	2.55	3.145 (2)	130
O3—H3*A*···O2	0.82	1.81	2.532 (2)	146
O3—H3*A*···O2^i^	0.82	2.38	2.856 (2)	118
C10—H10*A*···F2^ii^	0.93	2.51	3.326 (3)	147

Symmetry codes: (i) −*x*−1, −*y*+2, −*z*+1; (ii) −*x*−3, *y*+1/2, −*z*+1/2.

## References

[bb1] Baker, W. J. (1933). *J. Chem. Soc.* pp. 1381–1389.

[bb2] Bruker (2001). *SAINT*, *SMART* and *SADABS* Bruker AXS Inc., Madison, Wisconsin, USA.

[bb3] Fun, H.-K., Farhadikoutenaei, A., Narayana, B., Nayak, P. S. & Sarojini, B. K. (2012). *Acta Cryst.* E**68**, o2658. 10.1107/S1600536812034411 PMC3435684 22969555

[bb4] Mahal, H. S. & Venkataraman, K. (1934). *J. Chem. Soc.* pp. 1767–1769.

[bb5] Meng, C. Q., Ni, L. M., Worsencroft, K. J., Ye, Z., Weingarten, D. M., Simpson, J. E., Skudlarek, J. W., Marino, E. M., Suen, K. L., Kunsch, C., Souder, A., Howard, R. B., Sundell, C. L., Wasserman, M. A. & Sikorski, J. A. (2007). *J. Med. Chem.* **50**, 1304–1315. 10.1021/jm0614230 17323940

[bb6] Schobert, R., Biersack, B., Dietrich, A., Knauer, S., Zoldakova, M., Fruehauf, A. & Mueller, T. (2009). *J. Med. Chem.* **52**, 241–246. 10.1021/jm801001d 19102652

[bb7] Sheldrick, G. M. (2008). *Acta Cryst.* A**64**, 112–122. 10.1107/S0108767307043930 18156677

[bb8] Spek, A. L. (2009). *Acta Cryst.* D**65**, 148–155. 10.1107/S090744490804362X PMC2631630 19171970

